# Review of *Nitidotachinus* Campbell (Staphylinidae, Tachyporinae) from Mainland China

**DOI:** 10.3897/zookeys.447.8129

**Published:** 2014-10-16

**Authors:** Dan-Lin Zheng, Li-Zhen Li, Mei-Jun Zhao

**Affiliations:** 1 Department of Biology, College of Life and Environmental Sciences, Shanghai Normal University, 100 Guilin Road, Xuhui District, Shanghai 200234, P. R. China

**Keywords:** Coleoptera, *Nitidotachinus*, review, new species, new synonym, Mainland China

## Abstract

The genus *Nitidotachinus* Campbell of Mainland China is reviewed with descriptions of five new species: *Nitidotachinus anhuiensis* **sp. n.** (Anhui), *Nitidotachinus bini* **sp. n.** (Zhejiang), *Nitidotachinus brunneus* **sp. n.** (Zhejiang), *Nitidotachinus capillosus* **sp. n.** (Zhejiang), and *Nitidotachinus xiangi* **sp. n.** (Hubei). *Nitidotachinus excellens concolor* Schülke is synonymized with *Nitidotachinus excellens* **syn. n.** All treated species are described with their major diagnostic characters illustrated. An identification key to the species is given.

## Introduction

*Nitidotachinus* Campbell was established to hold the species formerly placed in the *Tachinus tachyporoides* and *Tachinus impunctatus* groups of *Tachinus* Gravenhorst (Campbell 1993) and *Tachinus excellens* Bernhauer. *Nitidotachinus tachyporoides* (Horn) was designated as the type species. Twelve species (including subspecies) are known worldwide, with four (including one species with doubtful records) from mainland China (Herman 2001, Smetana 2004, Schülke 2004).

*Nitidotachinus excellens* was the first Chinese species described as member of *Tachinus* by Bernhauer in 1938 from Manchuria, Hengtaohotze [now officially spelled as ‘Hengdaohezi, in Chinese: 横道河子’, located in Heilongjiang, Northeast China]. Later, *Nitidotachinus dui* Li, 1999 was described from Zhejiang and *Nitidotachinus excellens concolor* Schülke, 2000 was described from Sichuan.

*Nitidotachinus impunctatus* (Sharp) was originally described from Junsai Lake and Sapporo, Japan. With more collecting data published (Watanabe and Shibata 1961, Shibata 1985), the distribution of the species is now limited in Hokkaido and Honshu of Japan except few records from Jilin of Northeast China reported by Li and Chen (1990) and Li (1992, 1993). These Chinese records were published without any description or collecting datum, and many identification mistakes of other staphylinid groups are found in their papers. Thus, the distribution of *Nitidotachinus impunctatus* in China is highly doubtful, and thus this species is excluded from the present paper.

In the past few years, we collected a large series of *Nitidotachinus* specimens from Anhui, Hebei, Hubei, Sichuan and Zhejiang Provinces. Among this material, we recognized seven species from Mainland China, which includes five new species.

## Material and methods

Material used in this study is deposited in the Insect Collection of Shanghai Normal University, Shanghai, China (SNUC).

Habitus photos were taken using a Canon EOS 7D camera mounted with an MP-E 65 mm Macro Photo Lens. Photos of dissected parts were taken using a Canon G9 camera mounted on an Olympus CX21 microscope, and combined by the automontage software Zerene Stacker.

Total length was measured from the anterior margin of the head to the posterior margin of the abdominal tenth tergite; forebody length, from the anterior margin of the head to the posterior margin of the elytra; pronotum length, length of the pronotum along the midline; elytra length, length of the elytra along the suture; head width, width of the head across the eyes; pronotum width, maximum width of the pronotum.

## Systematics

### Nitidotachinus

Taxon classificationAnimaliaColeopteraStaphylinidae

Campbell, 1993

Nitidotachinus Campbell, 1993: 522; Downie and Arnett 1996: 470; Herman 2001: 849.

#### Type species.

*Tachinus tachyporoides* Horn, 1877.

#### Diagnosis.

Species of the genus are similar to those of allied genera *Tachinus* and *Leucotachinus*, but differ mainly in follows (Campbell 1993).

Body flatter, strongly shining. Ocular puncture obsolete; mentum with a pair of long apical setae and frequently a pair of basal setae; submentum setae; antennae elongate, with subapical segments at least 1.5 times as long as wide, with only first and second segments lacking dense pubescence; mandible narrowly elongate, with prostheca reduced, not extending beyond middle of mandible. Surface of pronotum almost impunctate, at most with minute fine punctures; mesocoxal cavities contiguous, with apex of mesosternal intercoxal process not reaching posteriorly to apex of metasternal intercoxal process. Abdominal tergites devoid of pruinose spots; posterior lateral corners of fouth–sixth tergites each with a long, projecting bristle. The empodial setae much longer than those of *Tachinus* and *Leucotachinus*.

#### Key to the species of *Nitidotachinus* from Mainland China

**Table:** 

1	Elytra with punctures and micropunctures; sixth sternite of male narrowly depressed medially, with curved row of short peg setae near apical margin; parameres of aedeagus longer than median lobe (the median lobe and parameres fused in *Nitidotachinus dui*), without long protuberance near apex. Eighth tergite of female with three pairs of long setae. (Because of the similarity in female sexual characters and the variation of female sexual characters within species, the following species can be precisely identified only by male characters.)	**2**
–	Elytra with micropunctures only (Fig. 1G); sixth sternite of male even medially, without short peg setae; parameres of aedeagus shorter than median lobe, each with a long protuberance near apex (Fig. 8A, G). Eighth tergite of female with four pairs of long setae	***Nitidotachinus excellens***
2	Median portion of seventh sternite with distinct pubescence in addition to areas of peg setae	**3**
–	Median portion of seventh sternite without pubescence, only with areas of peg setae in apical half	**5**
3	Seventh sternite with short peg setae area reaching the apical margin of sternite	**4**
–	Seventh sternite with short peg setae area distinctly separated from the apical margin of sternite	***Nitidotachinus xiangi* sp. n.**
4	Median portion of seventh sternite densely pubescent, peg setae area reaching anterior half of sternite	***Nitidotachinus capillosus* sp. n.**
–	Median portion of seventh sternite sparsely pubescent, peg setae area not reaching anterior half of sternite	***Nitidotachinus anhuiensis* sp. n.**
5	Aedeagus with parameres and median lobe fused entirely, parameres asymmetrical truncated at apex	***Nitidotachinus dui***
–	Aedeagus with parameres and median lobe separated, parameres symmetrical pointed at apex	**6**
6	Area of short peg setae on seventh sternite (Fig. 3B) smaller, about 1/6 times as long as entire sternite, and with posterior margin distinctly concave	***Nitidotachinus brunneus* sp. n.**
–	Area of short peg setae on seventh sternite (Fig. 7B) larger, about 1/3 times as long as entire sternite and with posterior margin slightly concave	***Nitidotachinus bini* sp. n.**

### Nitidotachinus capillosus sp. n.

Taxon classificationAnimaliaColeopteraStaphylinidae

http://zoobank.org/546F89C2-B07F-4165-B29F-5FF024F56DEB

Figs 1A , 2

#### Type specimens.

**Holotype:** **China:** male, Mt. Longwang Reserve (alt. 950–1200m), Anji County, Zhejiang Prov., 25.IV.2004, Jing-Wen Zhu leg. **Paratypes:** **China:** 3 males, same date and locality as holotype, Li-Long Zhu leg.; 1 male, same date and locality as holotype, Jing Chen leg.; 1 male, same date and locality as holotype, Shan-Jia Shen leg.; 2 females, same date and locality as holotype, Jin-Wen Li leg.

#### Description.

**Figure 1.**
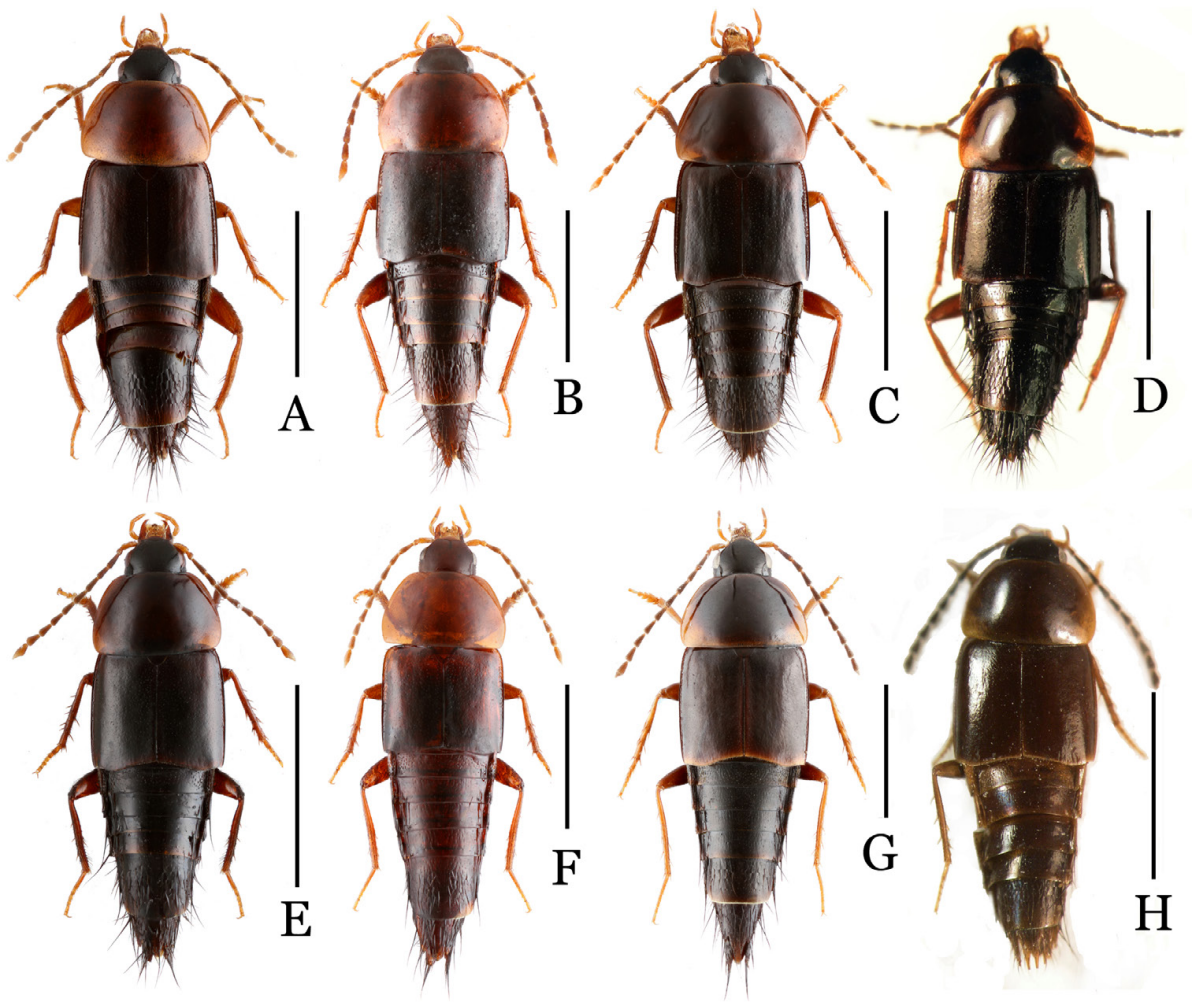
Habitus of *Nitidotachinus* spp. **A** *Nitidotachinus capillosus* sp. n. **B** *Nitidotachinus brunneus* sp. n. **C** *Nitidotachinus dui* **D** *Nitidotachinus xiangi* sp. n. **E** *Nitidotachinus anhuiensis* sp. n. **F** *Nitidotachinus bini* sp. n. **G** *Nitidotachinus excellens* **H** *Nitidotachinus excellens concolor* (paratype). Scale: 2 mm.

Body (Fig. 1A) medium in size, 4.2–5.2 mm (total length); 2.8–2.9 mm (length of forebody). Color dark brown with shine; head black; first and second antennal segments, mouthparts, margins of pronotum, narrow apical margins of both elytra and abdominal segments, and legs yellowish red; disc of pronotum, third to apical antennal segments dark reddish brown.

Head subtriangular, 0.49 times as wide as pronotum; surface very finely and sparsely punctate, with dense and coarse microsculpture consisting mostly of irregular meshes and transverse wave lines. Antennae long, extending backward beyond the middle of elytra; first and second segments glabrous except for a few long setae, third to 11th densely pubescent; the relative length of each segment from base to apex: 11.0 : 6.0 : 14.0 : 11.0 : 13.0 : 13.0 : 13.0 : 12.5 : 12.0 : 11.0 : 17.0; 10th segment 1.69 times as long as wide. Maxillary palpus moderately long, relative lengths of 4th and 3rd segments: 15.0 : 8.5.

Pronotum broad, transverse, 0.66 times as long as wide, widest at basal third. Surface with dense and fine microsculpture consisting of transverse wave lines; punctures similar to those on head.

Elytra in sutural length 0.67 times as long as wide; 1.09 times as long as the median length of pronotum; sides gradually widened posteriad; apical margins sinuate; apical angles sharp. Surface with punctures and microsculpture similar to those on pronotum.

Abdomen gradually narrowed from base to apex. Surface with many long bristles which are becoming denser towards anal apex, finely and sparsely punctate and pubescent, with minute microsculpture only at sides of third tergite.

**Figure 2.**
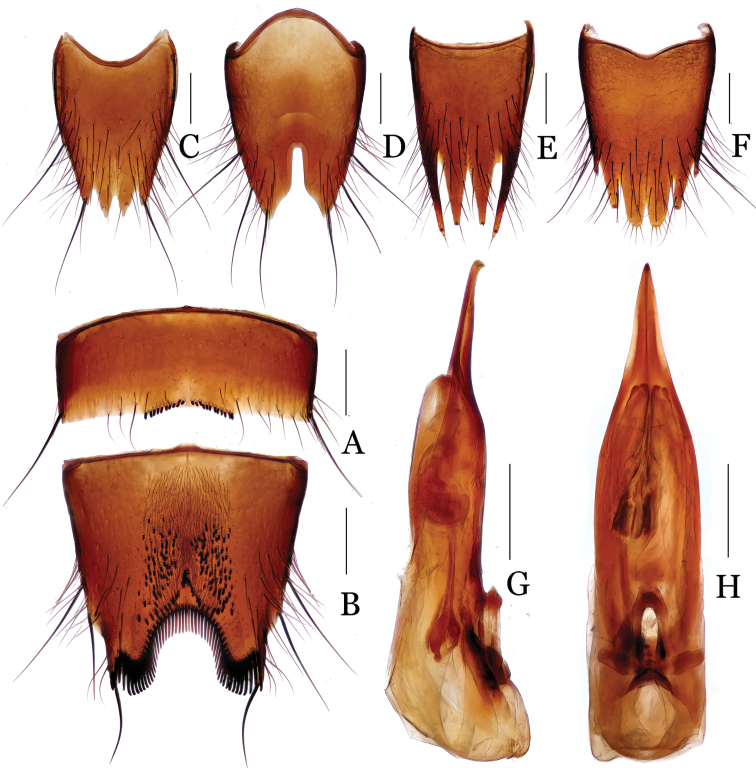
*Nitidotachinus capillosus* sp. n. **A** male 6th sternite **B** male 7th sternite **C** male 8th tergite **D** male 8th sternite **E** female 8th tergite **F** female 8th sternite **G** aedeagus in lateral view **H** aedeagus in ventral view. Scale: 0.3 mm.

Male: Fore tarsal segments I–IV dilated; the relative lengths of hind tarsal segments from base to apex: 14.0 : 5.0 : 4.0 : 4.0 : 9.0. Eighth tergite (Fig. 2C) 4-lobed; inner lobes separated from each other by a V-shaped emargination, much longer than outer lobes. Sixth sternite (Fig. 2A) slightly and arcuately emarginate at middle in apical margin, with 10–12 peg setae on each side of the emargination. Seventh sternite (Fig. 2B) subtriangularly depressed at middle in posterior part, roundly and deeply emarginate in apical margin, covered with a lot of peg setae and dense fine pubescence in the depression, densely bordered by long black spiniform setae on posterior margin. Eighth sternite (Fig. 2D) 2-lobed apically, deeply incised between the lobes, the depth 0.36 times as long as the length of sternite. Aedeagus (Figs 2G–H) with parameres much longer than median lobe, narrowed apicad and slightly curved ventrad near apices.

Female: Fore tarsal segments I–IV normal. Eighth tergite (Fig. 2E) 4-lobed; inner lobes distinctly shorter than outer lobes. Eighth sternite (Fig. 2F) 6-lobed; inner lobes much broader than intermediate lobes, separated from each other by a deep subtriangulate emargination.

#### Distribution.

China (Zhejiang Province).

#### Remarks.

This new species is similar to *Nitidotachinus anhuiensis* and *Nitidotachinus xiangi* by the median portion of male seventh sternite with distinct pubescence, but can be separated from *Nitidotachinus anhuiensis* by median portion of seventh sternite densely pubescent. It differs from *Nitidotachinus xiangi* by the male seventh sternite with short peg setae area reaching the apical margin of sternite.

#### Etymology.

The specific name is derived from a Latin word “capillosus” (= hairy), which refers to male seventh sternite with dense pubescence on disc.

### Nitidotachinus brunneus sp. n.

Taxon classificationAnimaliaColeopteraStaphylinidae

http://zoobank.org/719CF9A5-E3B5-46AA-9FC2-59491B99F87A

Figs 1B , 3

#### Type specimens.

**Holotype:** **China:** male, Mt. Longwang Reserve (alt. 950–1200m), Anji County, Zhejiang Prov., 25.IV.2004, Li-Long Zhu leg. **Paratypes:** **China:** 1 female, same data as holotype; 2 males, same locality as holotype, Liang Tang leg.; 1 male, 1 female, same locality as holotype, Jia-Jie Huang leg.; 1 male, same locality as holotype, Jia-Yao Hu leg.; 1 male, same locality as holotype, 25.IV.2006, Liang Tang leg.; 1 female, same locality as holotype, 25.IV.2006, Shan-Jia Shen leg.

#### Description.

Body (Fig. 1B) medium in size, 5.4–5.7 mm (total length); 2.9–3.2 mm (length of forebody). Color dark reddish brown with shine; first and second segments of antennae, mouthparts, sides of pronotum, and legs dark red.

Head subtriangular, 0.47 times as wide as pronotum. Surface finely and sparsely punctate, with dense and coarse microsculpture consisting mostly of irregular meshes. Antennae moderately long, reaching the apical third of elytra; 1st and 2nd segments glabrous except for a few long setae, 3rd to 11th densely pubescent; the relative length of each segment from base to apex: 13.0 : 8.0 : 16.0 : 13.0 : 14.0 : 15.0 : 14.0 : 13.0 : 13.0 : 13.0 : 17.0; the 10th segment 1.86 times as long as wide. Maxillary palpus moderately long, relative lengths of 4th and 3rd segments: 16.5 : 9.0.

Pronotum broad, transverse, 0.64 times as long as wide, widest at basal third. Surface with dense and fine microsculpture consisting of transverse wave lines, punctures slightly sparser and finer than those on head.

Elytra in sutural length 0.69 times as long as wide; 1.06 times as long as the median length of pronotum; sides gradually widened posteriad; apical margins sinuate; apical angles sharp. Surface with punctures much coarser than, and microsculpture similar to those on pronotum.

Abdomen gradually narrowed from base to apex. Surface with many long bristles which are becoming denser towards anal apex, very finely and sparsely punctate and pubescent, with minute microsculpture only at sides of third tergite.

**Figure 3.**
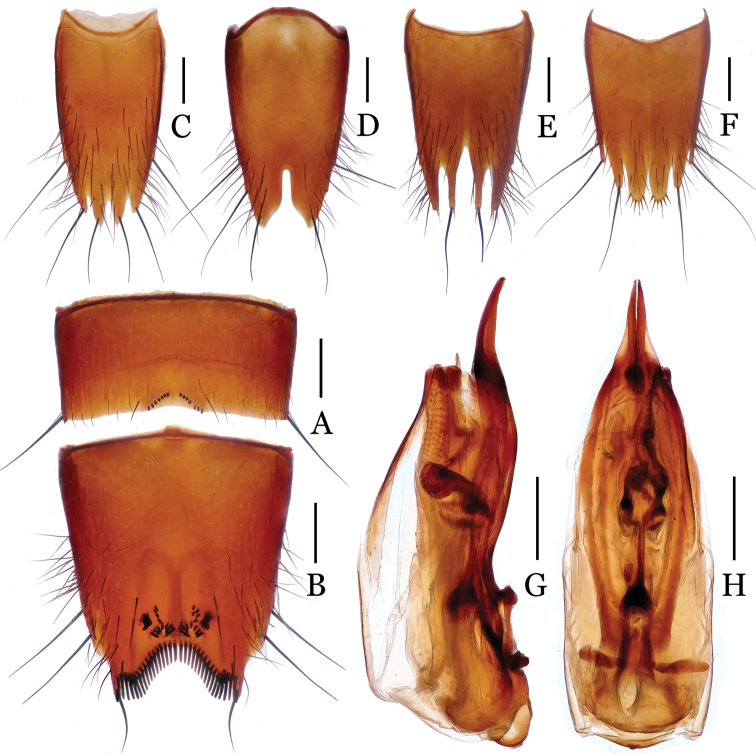
*Nitidotachinus brunneus* sp. n. **A** male 6th sternite **B** male 7th sternite **C** male 8th tergite **D** male 8th sternite **E** female 8th tergite **F** female 8th sternite **G** aedeagus in lateral view **H** aedeagus in ventral view. Scale: 0.3 mm.

Male: Fore tarsal segments I–IV dilated; the relative lengths of hind tarsal segments from base to apex: 13.0 : 6.0 : 4.5 : 4.0 : 10.0. Eighth tergite (Fig. 3C) 4-lobed; inner lobes separated from each other by a V-shaped emargination and much longer than outer lobes. Sixth sternite (Fig. 3A) slightly emarginate at middle in apical margin, with 7–8 granules on each side of the emargination. Seventh sternite (Fig. 3B) subtriangularly depressed at middle in posterior part, deeply and sinuately emarginated in apical margin, symmetrically covered with some short peg setae near posterior margin; densely bordered by long black spiniform setae on posterior margin. Eighth sternite (Fig. 3D) 2-lobed, deeply incised between two lobes, the depth 0.28 times as long as the median length of sternite; Aedeagus (Figs 3G–H) moderate in size; parameres longer than median lobe, strongly narrowed apicad, slightly curved ventrad in apical fourth.

Female: Fore tarsal segments I–IV normal. Eighth tergite (Fig. 3E) 4-lobed; inner lobes deeply separated with each other, much narrower and slightly shorter than outer lobes. Eighth sternite (Fig. 3F) 6-lobed; inner lobes much broader than intermediate lobes, separated from each other by a shallow, “V” shaped emargination.

#### Distribution.

China (Zhejiang Province).

#### Remarks.

This new species can be easily separated from the other species from Mainland China by the area of short peg setae on male seventh sternite smaller, about 1/6 times as long as entire sternite.

#### Etymology.

The specific name is derived from a Latin word “brunneus” (= brown), which refers to the body color.

### Nitidotachinus dui

Taxon classificationAnimaliaColeopteraStaphylinidae

Li, 1999

Figs 1C , 4

Nitidotachinus dui Li, 1999: 197; Schülke 2000: 907; Herman 2001: 850; Smetana 2004: 340.

#### Specimens examined.

**China:** 1 male (holotype), 1 female (paratype), Mt. West Tianmu Reserve, Lin’an City, Zhejiang Prov., 6–12.V.1998, Li-Zhen Li leg.; 1 male, 8 females, same locality as above, (alt. 300–400m), 11–15.VI.2006, Jia-Yao Hu and Liang Tang leg.; 1 female, Mt. Longwang Reserve (alt. 300–500m), Anji County, Zhejiang Prov., 24.IV.2004, Liang Tang leg.; 4 males, 7 females, Danzhu (alt. 450–600m), Xianju County, Zhejiang Prov., 2.VI.2006, Jin-Wen Li and Shan-Jia Shen leg.; 1 male, Mt. Dapan Reserve (alt. 550–700m), Pan’an County, Zhejiang Prov., 7.VI.2006, Jin-Wen Li and Shan-Jia Shen leg.; 3 female, same locality and collectors as above, 6.VI.2006; 1 female, Qingliangfeng (alt. 1050–1070m), Lin’an City, Zhejiang Prov., 9.V.2005, Li-Long Zhu and Li-Zhen Li leg.

#### Description.

Body (Fig. 1C) relative small in size, 4.8–5.0 mm (total length); 2.6–2.8 mm (length of forebody). Color piceous, shining; head black; 1st and 2nd antennal segments, mouthparts, lateral margins of pronotum, narrow apical margins of abdominal segments, and tarsi light reddish brown; disc of pronotum, 3rd to 11th antennal segments, and legs except for tarsi dark reddish brown.

Head subtriangular, 0.48 times as wide as pronotum; surface finely and sparsely punctate, with dense microsculpture consisting of irregular meshes and transverse wave lines. Antennae moderately long, reaching backward to the apical third of elytra; 1st and 2nd segments glabrous except for a few long setae, 3th to 11th densely pubescent; the relative length of each segment from base to apex: 12.0 : 7.0 : 17.0 : 12.0 : 15.0 : 14.0 : 14.0 : 13.0 : 13.0 : 12.5 : 17.0; the 10th segment twice as long as wide. Maxillary palpus moderately long, relative lengths of 4th and 3rd segments: 2.0 : 1.0.

Pronotum broad, transverse, 0.63 times as long as wide, widest at basal third. Surface with dense and fine microsculpture consisting of transverse wave lines; punctures similar to those on head.

Elytra in sutural length 0.73 times as long as wide; 1.17 times as long as the median length of pronotum; sides gradually widened posteriad; apical margins sinuate. Surface with punctures courser and microsculpture somewhat finer than those on pronotum.

Abdomen gradually narrowed from base to apex. Surface sparsely and finely punctate and pubescent, with short transverse microsculpture only at sides of third tergite.

**Figure 4.**
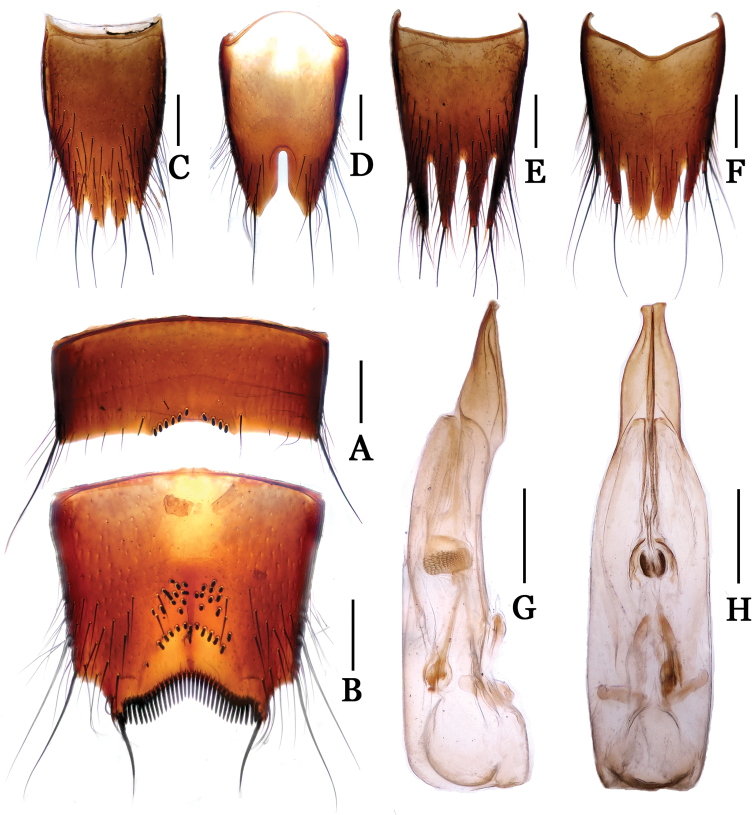
*Nitidotachinus dui*. **A** male 6th sternite **B** male 7th sternite **C** male 8th tergite **D** male 8th sternite **E** female 8th tergite **F** female 8th sternite **G** aedeagus in lateral view **H** aedeagus in ventral view. Scale: 0.3 mm.

Male: Fore tarsal segments I–IV dilated; the relative lengths of hind tarsal segments from base to apex: 14.0 : 5.0 : 4.0 : 4.0 : 9.0. Eighth tergite (Fig. 4C) 4-lobed; inner lobes much longer than outer lobes. Sixth sternite (Fig. 4A) arcuately emarginate at middle in apical margin, with nine peg setae on each side of the emargination. Seventh sternite (Fig. 4B) subtriangularly depressed at middle in posterior part, deeply and sinuately emarginated in apical margin, sparsely covered with some peg setae near middle of the subtriangular depression, densely bordered by long black spiniform setae on posterior margin. Eighth sternite (Fig. 4D) 2-lobed, deeply incised between the lobes, the depth 0.33 times as long as the median length of sternite. Aedeagus (Figs 4G–H) moderate in size, with parameres and median lobe fused, asymmetrical, narrowed apicad, distinctly widened and truncated at apices in ventral view.

Female: Fore tarsal segments 1–4 normal. Eighth tergite (Fig. 4E) 4-lobed; inner lobes slightly longer than outer lobes. Eighth sternite (Fig. 4F) 6-lobed; inner lobes much broader than intermediate lobes, fimbriate apically, separated from each other by a “V” shaped emargination.

#### Distribution.

China (Zhejiang Province).

#### Remarks.

This species can be easily recognized from the others of the genus by parameres and median lobe of aedeagus being fused and the asymmetrical truncated apices of parameres.

### Nitidotachinus xiangi sp. n.

Taxon classificationAnimaliaColeopteraStaphylinidae

http://zoobank.org/4D76772A-2D99-4167-B374-20B6050D8E1B

Figs 1D , 5

#### Type specimens.

**Holotype:** **China:** male, Houhe Conv., Wufeng County, Hubei Prov., 1.V.2004, Li-Zhen Li leg. **Paratypes:** **China:** 1 female, same locality as holotype, 30.IV.2004, Li-Zhen Li leg.

#### Description.

Body (Fig. 1D) relative large in size, 5.7–6.0 mm (total length); 2.9–3.1 mm (length of forebody). Color dark reddish brown with shine; head black; first and second antennal segments, mouthparts, lateral margins of pronotum, narrow apical margins of both elytra and abdominal segments, and legs yellowish red; disc of pronotum, 3rd to apical segments of antennae reddish brown.

Head subtriangular, 0.48 times as wide as pronotum; surface finely and sparsely punctate, with dense microsculpture consisting mostly of transverse wave lines. Antennae long, extending backward beyond the middle of elytra; 1st and 2nd segments glabrous except for a few setae, 3rd to 11th densely pubescent; the relative length of each segment from base to apex: 11.5 : 7.0 : 16.0 : 12.0 : 14.0 : 14.0 : 13.0 : 13.0 : 13.0 : 13.0 : 16.5; the 10th segment 1.71 times as long as wide. Maxillary palpus moderately long, relative lengths of 4th and 3rd segments: 16.0 : 9.0.

Pronotum broad, transverse, 0.66 times as long as wide, widest at basal third. Surface with dense and fine microsculpture consisting of transverse wave lines; punctures similar to those on head.

Elytra in sutural length 0.62 times as long as wide; 1.02 times as long as the median length of pronotum; sides gradually widened posteriad; apical margins sinuate; apical angles sharp. Surface with punctures much courser, and microsculpture somewhat finer than those on pronotum.

Abdomen gradually narrowed from base to apex. Surface with many long bristles which are becoming denser towards apex, finely and sparsely punctate and pubescent, with minute microsculpture only at sides of third tergite.

**Figure 5.**
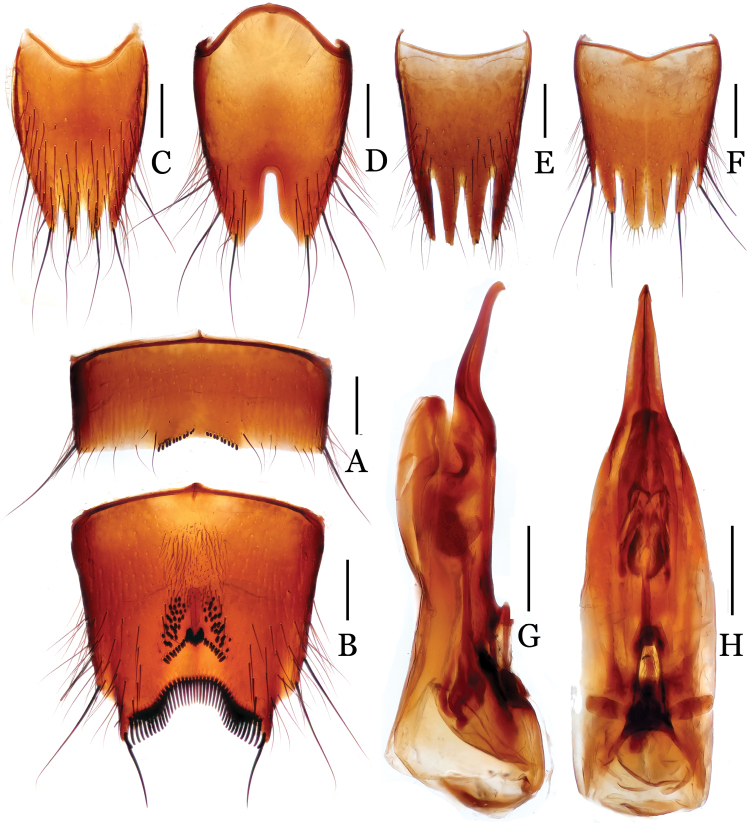
*Nitidotachinus xiangi* sp. n. **A** male 6th sternite **B** male 7th sternite **C** male 8th tergite **D** male 8th sternite **E** female 8th tergite **F** female 8th sternite **G** aedeagus in lateral view **H** aedeagus in ventral view. Scale: 0.3 mm.

Male: Fore tarsal segments I–IV dilated; the relative lengths of hind tarsal segments from base to apex: 14.5 : 6.0 : 4.5 : 4.0 : 16.0. Eighth tergite (Fig. 5C) 4-lobed; inner lobes separated from each other by a V-shaped emargination, much longer than outer lobes. Sixth sternite (Fig. 5A) slightly and roundly emarginate at middle in apical margin, with 9–11 peg setae on each side of the emargination. Seventh sternite (Fig. 5B) subtriangularly depressed at middle in posterior part, deeply and sinuately emarginated in apical margin, symmetrically covered with a lot of short peg setae in median part of the depression, densely pubescent in the depression before the area of peg setae, and densely bordered by long black spiniform setae on posterior margin. Eighth sternite (Fig. 5D) 2-lobed apically, deeply incised between the lobes, the depth 0.32 times as long as the median length of sternite. Aedeagus (Figs 5G–H) somewhat elongate; parameres much longer than median lobe, arrowhead-shaped at apices in ventral view, slightly curved ventrad at apical third.

Female: Fore tarsal segments I–IV normal. Eighth tergite (Fig. 5E) 4-lobed; inner lobes slightly longer than outer lobes. Eighth sternite (Fig. 5F) 6-lobed; inner lobes much broader than intermediate lobes, distinctly separated from each other.

#### Distribution.

China (Hubei Province).

#### Remarks.

This new species is similar to *Nitidotachinus capillosus* and *Nitidotachinus anhuiensis*. But it can be easily separated from them by the male seventh sternite with short peg setae area distinctly separated from the apical margin of sternite.

#### Etymology.

The specific name is derived from the name of Prof. Jia-Xiang Xiang, the vice-president of Shanghai Normal University, who helped us in many ways during our studies.

### Nitidotachinus anhuiensis sp. n.

Taxon classificationAnimaliaColeopteraStaphylinidae

http://zoobank.org/E674BDE3-3C5B-434D-984E-87D9C40957BC

Figs 1E , 6

#### Type specimens.

**Holotype:** **China:** male, Mt. Tianzhu (alt. 960m), Anhui Prov., 23.IV.2005, Jia-Yao Hu and Liang Tang leg. **Paratypes:** **China:** 1 male, 4 females, same data as holotype.

#### Description.

Body (Fig. 1E) medium in size, 4.5–5.7 mm (total length); 2.8–3.2 mm (length of forebody). Color dark brown with shine; the first and second segments of antennae and mouthparts, sides of pronotum, posterior margins of elytra, and legs reddish brown.

Head subtriangular, 0.48 times as wide as pronotum. Surface finely and sparsely punctate, with dense and coarse microsculpture consisting of irregular meshes and transverse wave lines. Antennae moderately long, reaching the apical third of elytra; 1st and seconf segments glabrous except for a few long setae, 3rd to 11th densely pubescent; the relative length of each segment from base to apex: 11.5 : 7.0 : 14.0 : 10.5 : 12.0 : 12.0 : 12.0 : 11.5 : 11.5 : 11.5 : 15.0; the 1tenth segment 1.92 times as long as wide. Maxillary palpus moderately long, relative lengths of 4th and 3rd segments: 15.5 : 9.0.

Pronotum broad, transverse, 0.65 times as long as wide, widest at basal third. Surface with microsculpture finer and punctures slightly sparser and shallower than those on head.

Elytra in sutural length 0.68 times as long as wide; 1.10 times as long as the median length of pronotum; sides gradually widened posteriad; apical margins sinuate; apical angles sharp. Surface with punctures coarser than, and microsculpture similar to those on pronotum.

Abdomen gradually narrowed from base to apex. Surface with many long bristles which are becoming denser towards anal apex, very finely and sparsely punctate and pubescent, with minute microsculpture only at sides of third tergite.

**Figure 6.**
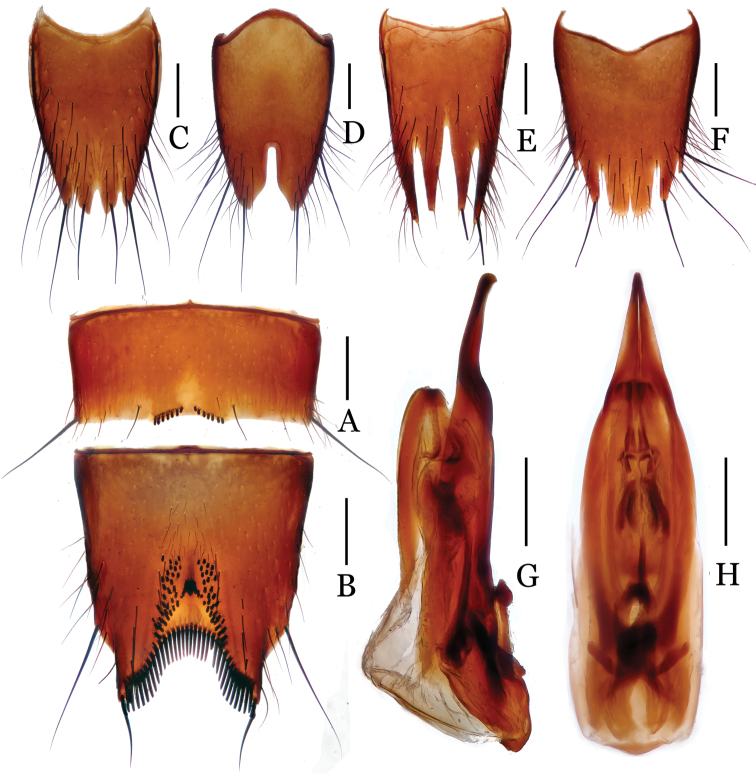
*Nitidotachinus anhuiensis* sp. n. **A** male 6th sternite **B** male 7th sternite **C** male 8th tergite **D** male 8th sternite **E** female 8th tergite **F** female 8th sternite **G** aedeagus in lateral view **H** aedeagus in ventral view. Scale: 0.3 mm.

Male: Fore tarsal segments I–IV dilated; the relative lengths of hind tarsal segments from base to apex: 14.0 : 5.0 : 4.5 : 3.5 : 9.0. Eighth tergite (Fig. 6C) 4-lobed; inner lobes separated from each other by a V-shaped emargination and distinctly longer than outer lobes. Sixth sternite (Fig. 6A) slightly emarginate at middle in apical margin, with seven short peg setae on each side of the emargination. Seventh sternite (Fig. 6B) subtriangularly depressed at middle in posterior part, very deeply and sinuately emarginated at the middle in apical margin, symmetrically covered with many short peg setae in apical half and fine setae in basal half of the depression respectively; densely covered with long black spiniform setae on posterior margin. Eighth sternite (Fig. 6D) 2-lobed, deeply incised between two lobes, the depth 0.31 times as long as the median length of sternite. Aedeagus (Figs 6G–H) moderately long, with parameres longer than median lobe, narrowed apicad and slightly curved ventrad at apical portions.

Female: Fore tarsal segments I–IV normal. Eighth tergite (Fig. 6E) 4-lobed; inner lobes deeply separated from each other, distinctly shorter than outer lobes. Eighth sternite (Fig. 6F) 6-lobed; inner lobes much broader than intermediate lobes, separated from each other by a shallow, “V” shaped emargination.

#### Distribution.

China (Anhui Province).

#### Remarks.

This new species is similar to *Nitidotachinus capillosus* and *Nitidotachinus xiangi*. But can be easily separated from *Nitidotachinus capillosus* by the median portion of male seventh sternite sparsely pubescent. It differs from *Nitidotachinus xiangi* by the male seventh sternite with short peg setae area reaching the apical margin of sternite.

#### Etymology.

The specific name is named after Anhui Province, where the type specimens were collected.

### Nitidotachinus bini sp. n.

Taxon classificationAnimaliaColeopteraStaphylinidae

http://zoobank.org/074B0938-76AB-4902-BC5F-59501D3FE5A7

Figs 1F , 7

#### Type specimens.

**Holotype:** **China:** male, Qingliangfeng Reserve (alt.1080m), Zhejiang Prov., 10.V.2005, Li-Long Zhu & Li-Zhen Li leg. **Paratypes:** **China:** 3 males, 1 female, same data as holotype, but data from 8–10.V.2005; 1male, 2 females, Mt. Dapan Reserve (alt.550–800m), Pan’an County, Zhejiang Prov. 6–7.VI.2006, Li-Zhen Li & Shan-jia Shen leg.

#### Description.

*Male* (Fig. 1F): Body medium in size, 5.7–6.0 mm (total length); 2.8–2.9 mm; 2.5–2.8 mm (length of forebody). Color reddish brown with shine; head black, the 1st and 2nd segments of antennae, mouthparts, sides of pronotum, posterior margins of elytra, and legs light reddish brown.

Head 0.47 times as wide as pronotum. Surface finely and sparsely punctate, with dense and coarse microsculpture consisting of irregular meshes and transverse wave lines. Antennae long, reaching the apical third of elytra; the relative length of each segment from base to apex: 13.0 : 8.0 : 16.0 : 11.0 : 13.0 : 13.0 : 13.0 : 13.0 : 13.0 : 12.0 : 17.0; the 10th segment 1.60 times as long as wide. Maxillary palpus with relative lengths of 4th and 3rd segments: 16.5 : 9.0.

Pronotum 0.63 times as long as wide; microsculpture shallower and punctures slightly sparser than those on head.

Elytra in sutural length 0.67 times as long as wide; 1.05 times as long as the median length of pronotum; punctures coarser than, and microsculpture similar to those on pronotum.

Abdomen sparsely and finely punctate and pubescent, with minute microsculpture only at sides of third tergite.

**Figure 7.**
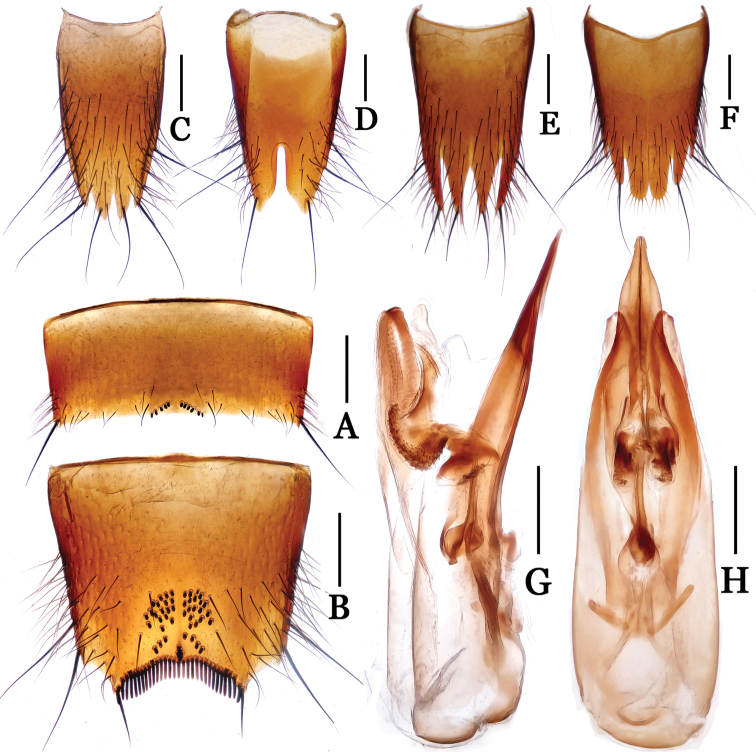
*Nitidotachinus bini* sp. n. **A** male 6th sternite **B** male 7th sternite **C** male 8th tergite **D** male 8th sternite **E** female 8th tergite **F** female 8th sternite **G** aedeagus in lateral view **H** aedeagus in ventral view. Scale: 0.3 mm.

Male: Fore tarsal segments I–IV dilated; the relative lengths of hind tarsal segments from base to apex: 15.0 : 5.0 : 4.5 : 4.0 : 9.0. Eighth tergite (Fig. 7C) 4-lobed; inner lobes separated from each other by a V-shaped emargination, much longer than outer lobes. Sixth sternite (Fig. 7A) slightly emarginate at middle in apical margin, with 6–8 short peg setae on each side of the emargination. Seventh sternite (Fig. 7B) subtriangularly depressed at middle in posterior part, roundly emarginated at middle in apical margin, symmetrically covered with some short peg setae in apical half of the depression, densely covered with long black spiniform setae on apical margin. Eighth sternite (Fig. 7D) 2-lobed, deeply incised between two lobes, the depth 0.30 times as long as the median length of sternite. Aedeagus (Figs 7G–H) with parameres longer than median lobe, tapered apicad.

Female: Fore tarsal segments I–IV normal. Eighth tergite (Fig. 7E) 4-lobed; inner lobes deeply separated with each other, about as long as outer lobes. Eighth sternite (Fig. 7F) 6-lobed; inner lobes distinctly separated from each other.

#### Distribution.

China (Zhejiang Province).

#### Remarks.

This new species is similar to *Nitidotachinus taiwanensis*. But can be separated from it by the shape of peg setae area of male seventh sternite; female eighth tergite with emargination between the inner lobes is distinctly shallower than those between inner and outer lobes; and parameres of aedeagus a little narrower in apical parts.

#### Etymology.

The specific name is named after the nick name of Xiao-bin Song.

### Nitidotachinus excellens

Taxon classificationAnimaliaColeopteraStaphylinidae

(Bernhauer, 1938)

Figs 1G–H , 8 , 9

Tachinus excellens Bernhauer, 1938: 23; Li and Chen 1990: 19; Li 1992: 55.
Tachinus exceliens : Li 1993: 43.
Tachinus sawadai Watanabe & Shibata, 1961: 36; Campbell 1993: 531.
Nitidotachinus excellens : Campbell 1995: 45; Herman 2001: 850; Smetana 2004: 340; Schülke 2005: 170.
Nitidotachinus excellens concolor Schülke, 2000: 907. **syn. n.**

#### Specimens examined.

**China:** 5 males, 5 females Mt. Xiaowutai Reserve (alt. 1300–1600m), Wei County, Hebei Prov., 22.VIII.2005, Li-Zhen Li leg.; 1 male, 1 female, Jinhekou (alt. 1300m), Wei County, Hebei Prov., 23.VIII.2005, Li-Zhen Li leg.; 11 males, 10 females, Labahe Reserve (alt. 1900m), Tianquan County, Sichuan Prov., 29.VII.2006, Jia-Yao Hu and Liang Tang leg.; 1 male, 5 females, Labahe Reserve (alt. 2000m), Tianquan County, Sichuan Prov., 30.VII.2006, Jia-Yao Hu and Liang Tang leg.; 1 males, 1 females, Foping Conv. (alt. 1400–1800m), Shaanxi Prov., 19.VII.2004, Jia-Yao Hu, Liang Tang and Li-Long Zhu leg.; 1 male, ditto, but (alt.1250–1400m), 18.VII.2004; 1 female, Yangjiaping (alt.830m), Zhulu Couty, Hebei Prov., 4.VIII.2005, Li-Zhen Li leg.; 1 female (paratype), with labels as: CHINA: S-Sichuan 1999, Ya’an Prefecture, Shimian Co., Xiaoxiang Ling, Pass zw. Shimian u., Ganluo, 27 km SE Shimian, 29°03N, 102°31E, 2450m, Quellsumpf, Bachufer, 8. VII., leg. M/ Schülke (white) / Sammlung M. Schülke, Berlin (green) / PARATYPUS, Nitidotachinus exellens subspec. concolor n., det. M. Schülke, 2000 (red).

#### Description.

Body (Fig. 1G–H) medium in size, 4.5–5.9 mm (total length); 3.1–3.4 mm (length of forebody). Color dark brown with shine; the first and second segments of antennae, mouthparts, sides and posterior margin of pronotum, posterior margins of elytra, and legs yellowish red.

Head 0.48 times as wide as pronotum. Surface finely and sparsely punctate, with dense and coarse microsculpture consisting of irregular meshes and transverse wave lines. Antennae moderately long, reaching the middle of elytra; the relative length of each segment from base to apex: 11.5 : 7.0 : 15.0 : 12.0 : 14.0 : 14.5 : 13.0 : 13.0 : 13.0 : 13.0 : 16.0; the tenth segment 1.73 times as long as wide. Maxillary palpus with relative lengths of 4th and 3rd segments: 15.5 : 8.0.

Pronotum 0.65 times as long as wide; microsculpture shallower and punctures much finer and sparser than those on head.

Elytra in sutural length 0.72 times as long as wide; 1.15 times as long as the median length of pronotum; punctures and microsculpture a little courser than those on pronotum.

Abdomen sparsely and finely punctate and pubescent, with minute microsculpture only at sides of third tergite.

**Figure 8.**
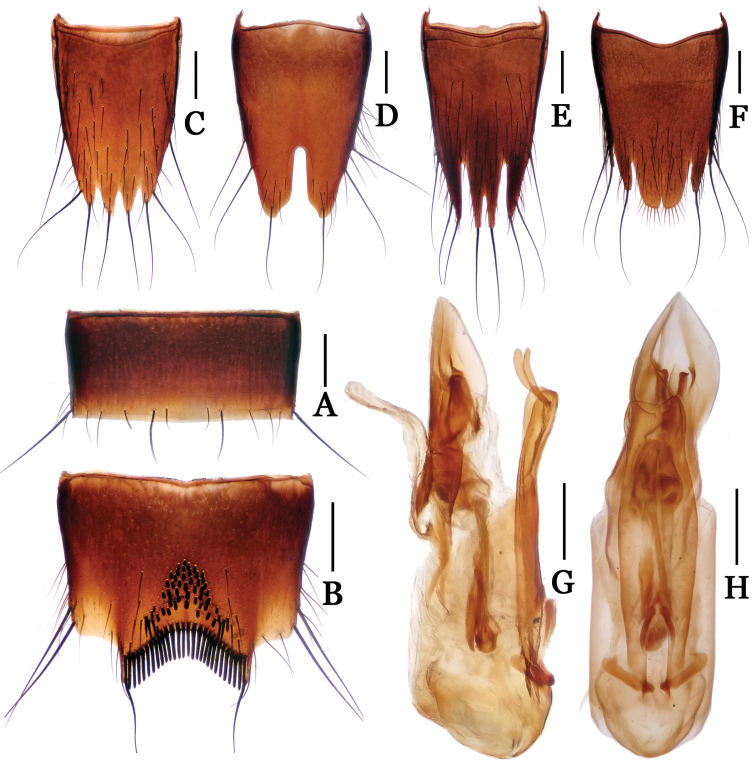
*Nitidotachinus excellens*. **A** male 6th sternite **B** male 7th sternite **C** male 8th tergite **D** male 8th sternite **E** female 8th tergite **F** female 8th sternite **G** aedeagus in lateral view **H** aedeagus in ventral view. Scale: 0.3 mm.

Male: Fore tarsal segments I–IV dilated; the relative lengths of hind tarsal segments from base to apex: 14.0 : 7.5 : 5.0 : 4.0 : 10.0. Eighth tergite (Fig. 8C) 4-lobed; inner lobes separated from each other by a V-shaped emargination and distinctly longer than outer lobes. Sixth sternite (Fig. 8A) without short peg seta. Seventh sternite (Fig. 8B) subtriangularly depressed at middle in posterior part, roundly emarginated at middle in apical margin, symmetrically covered with sparse short peg setae in the depression before posterior margin, densely covered with long black spiniform setae on posterior margin. Eighth sternite (Fig. 8D) 2-lobed, deeply incised between two lobes, the depth 0.35 times as long as the median length of sternite. Aedeagus (Figs 8G–H) medium in size; parameres shorter than median lobe, each with a spindly projection near apex.

**Figure 9.**
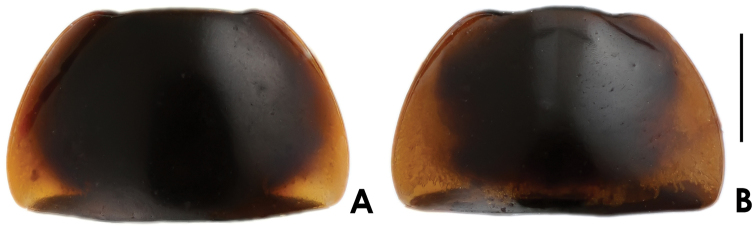
*Nitidotachinus excellens*. **A** Pronotum, specimen collected from Labahe, Tianquan, Ya’an, Sichuan **B** ditto. Scale: 0.5 mm.

Female (Fig. 8): Fore tarsal segments I–IV normal. Eighth tergite (Fig. 8E) 4-lobed; inner lobes relatively shallowly separated with each other, distinctly longer than outer lobes. Eighth sternite (Fig. 8F) 6-lobed; inner lobes almost fused with each other, slightly emarginate at middle in posterior margins.

#### Distribution.

China (Hebei, Shaanxi, Sichuan, Heilongjiang, Jilin, Liaoning, Beijing); Japan; Far East Russia; Korea.

**Figure 10.**
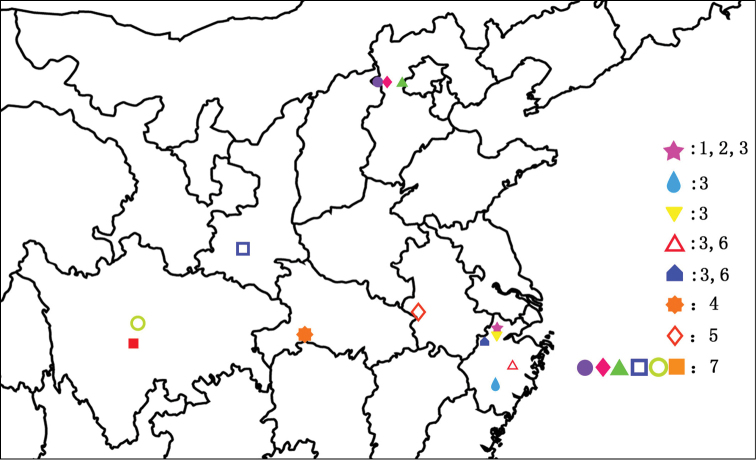
Distribution of *Nitidotachinus* species from Mainland China: **1** *Nitidotachinus capillosus* sp. n. **2** *Nitidotachinus brunneus* sp. n. **3** *Nitidotachinus dui* **4** *Nitidotachinus xiangi* sp. n. **5** *Nitidotachinus anhuiensis* sp. n. **6** *Nitidotachinus bini* sp. n. **7** *Nitidotachinus excellens*.

#### Remarks.

This new species is similar to *Nitidotachinus impunctatus* and *Nitidotachinus japonicus*. But can be easily separated from them by the different shape of aedeagal parameres.

#### Comments.

*Nitidotachinus excellens concolor* was described by Schülke in 2000 from Xiaoxiang Ling, Shimian, Ya’an, Sichuan Province. The characters separating it from the nominal subspecies were mentioned to be almost total black pronotum, darker elytra and inner emargination of female eighth tergite slightly deeper and wider. Though a paratype (Fig. 1H) of it also shows the broad yellowish brown lateral margins on pronotum. With more material collected in recent years, those diagnosis characters mentioned above are now considered as variability within *Nitidotachinus excellens* and the distribution gap between two subspecies are totally filled. Thus *Nitidotachinus excellens concolor* has to be synonymized with *Nitidotachinus excellens*, which is also fully agreed by M. Schülke (personal communication).

## Supplementary Material

XML Treatment for Nitidotachinus

XML Treatment for Nitidotachinus capillosus

XML Treatment for Nitidotachinus brunneus

XML Treatment for Nitidotachinus dui

XML Treatment for Nitidotachinus xiangi

XML Treatment for Nitidotachinus anhuiensis

XML Treatment for Nitidotachinus bini

XML Treatment for Nitidotachinus excellens
